# Altered Local Interactions and Long-Range Communications in UK Variant (B.1.1.7) Spike Glycoprotein

**DOI:** 10.3390/ijms22115464

**Published:** 2021-05-22

**Authors:** Stefano Borocci, Carmen Cerchia, Alessandro Grottesi, Nico Sanna, Ingrid Guarnetti Prandi, Nabil Abid, Andrea R. Beccari, Giovanni Chillemi, Carmine Talarico

**Affiliations:** 1 Department for Innovation in Biological, Agro-Food and Forest Systems, DIBAF, University of Tuscia, Via S. Camillo de Lellis s.n.c., 01100 Viterbo, Italy; borocci@unitus.it (S.B.); n.sanna@unitus.it (N.S.); 2 Institute for Biological Systems, ISB, CNR, Via Salaria, Km 29.500, Monterotondo, 00015 Rome, Italy; 3 Department of Pharmacy, University of Napoli “Federico II”, Via D. Montesano 49, 80131 Napoli, Italy; carmen.cerchia@unina.it; 4 Department HPC, CINECA, Via dei Tizii 6, 00185 Roma, Italy; a.grottesi@cineca.it; 5 Department of Chemistry and Industrial Chemistry, University of Pisa, Via Giuseppe Moruzzi 3, 56124 Pisa, Italy; ingrid.prandi@gmail.com; 6 Laboratory of Transmissible Diseases and Biological Active Substances LR99ES27, Faculty of Pharmacy, University of Monastir, Rue Ibn Sina, Monastir 5000, Tunisia; nabilabidbensalem.2014@yahoo.fr; 7 High Institute of Biotechnology of Sidi Thabet, Department of Biotechnology, University of Manouba, BP-66, Ariana-Tunis 2020, Tunisia; 8 Dompé Farmaceutici SpA, Via Campo di Pile, 67100 L’Aquila, Italy; andrea.beccari@dompe.com; 9 Institute of Biomembranes, Bioenergetics and Molecular Biotechnologies, IBIOM, CNR, Via Giovanni Amendola, 122/O, 70126 Bari, Italy

**Keywords:** SARS-CoV-2, COVID-19, spike, variants, molecular dynamics

## Abstract

The COVID-19 pandemic is caused by SARS-CoV-2. Currently, most of the research efforts towards the development of vaccines and antibodies against SARS-CoV-2 were mainly focused on the spike (S) protein, which mediates virus entry into the host cell by binding to ACE2. As the virus SARS-CoV-2 continues to spread globally, variants have emerged, characterized by multiple mutations of the S glycoprotein. Herein, we employed microsecond-long molecular dynamics simulations to study the impact of the mutations of the S glycoprotein in SARS-CoV-2 Variant of Concern 202012/01 (B.1.1.7), termed the “UK variant”, in comparison with the wild type, with the aim to decipher the structural basis of the reported increased infectivity and virulence. The simulations provided insights on the different dynamics of UK and wild-type S glycoprotein, regarding in particular the Receptor Binding Domain (RBD). In addition, we investigated the role of glycans in modulating the conformational transitions of the RBD. The overall results showed that the UK mutant experiences higher flexibility in the RBD with respect to wild type; this behavior might be correlated with the increased transmission reported for this variant. Our work also adds useful structural information on antigenic “hotspots” and epitopes targeted by neutralizing antibodies.

## 1. Introduction

Coronavirus disease 2019 (COVID-19), caused by severe acute respiratory syndrome coronavirus 2 (SARS-CoV-2), has quickly spread worldwide and has caused a global health crisis. Coronaviruses (CoVs) are lipid-enveloped positive-sense single-stranded RNA viruses (+ssRNA). The glycoprotein spike, (S), composes the viral envelope, together with other two structural proteins, the envelope (E) and membrane (M), whereas the nucleocapsid (N) protein binds and protects the (+)ss RNA genome inside the viral particle [1]. The glycoprotein S, which extensively decorates the viral envelope, ensures the recognition and fusion steps with the host cell, initiating the infection process [1]. Additionally, the S glycoprotein induces neutralizing antibody responses and thus represents a key target for vaccine development [2]. Among these vaccines, both Pfizer/BioNTech and Moderna use mRNA encoding for the S glycoprotein [3,4] while Gamaleya Sputnik V [5], Oxford/AstraZeneca [6], and CanSino [7] vaccines are based on adenoviruses, as vectors encoding for the full-length S glycoprotein.

**Figure 1:**
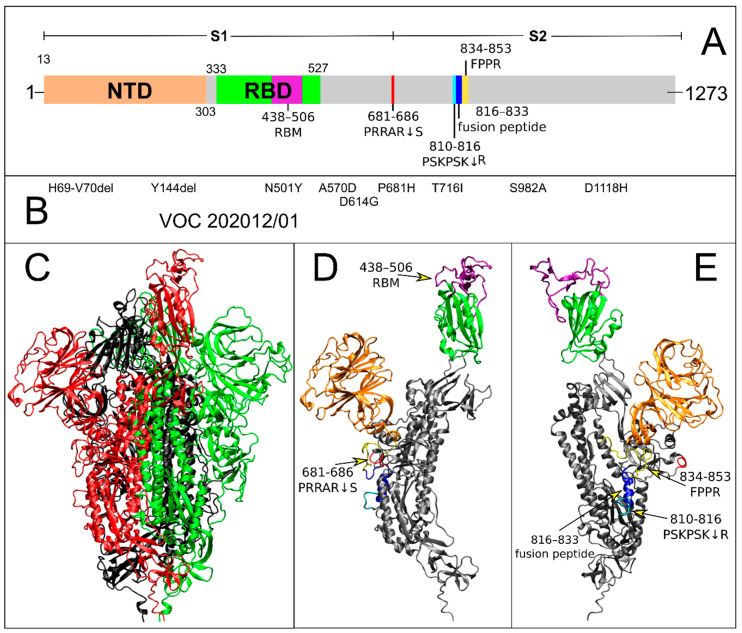
Domain organization and UK variant sequences in the S protein. (**A**) The S protein is divided in two regions, S1 and S2. In S1, the NTD and RBD are colored in orange and green, respectively. Within the RBD, the RBM is highlighted in purple. The newly acquired furin-like cleavage site at the S1/S2 boundary is highlighted in red. The two neighboring second cleavage site [35] and fusion peptide regions are highlighted in cyan and blue, respectively. (**B**) The VOC 202012/01 mutations are mapped along the S protein sequence. (**C**) Cartoon representation of the WT snapshot after 629.8 ns of MD simulation, centroid of the most populated protein cluster. The three monomers are colored in black, red, and green, respectively. Monomer 2 is the one in up conformation. (**D**,**E**) Monomer 2 of the same MD snapshot in two different orientations. S protein key regions are colored and highlighted as in panel A.

From the structural point of view, the S glycoprotein is about 1200 aa long, homotrimeric, class I fusion protein (Figure 1A). Each monomer comprises two subunits, S1 and S2. The S1 subunit mediates receptor binding and recognition, whereas the S2 subunit is responsible for virus–cell membrane fusion [8]. The S1 subunit contains an N-terminal domain (NTD) and the receptor binding domain (RBD), harboring a receptor binding motif (RBM), responsible for the early recognition step with the angiotensin-converting enzyme 2 (ACE2) receptor, enhancing the entry of the virus into the cell host [9]. The RBD can be found in two distinct conformations: “up”, a host receptor-accessible state and “down”, representing a host receptor-inaccessible state. The interaction interface in glycoprotein S/ACE2 complex has been elucidated by several recently published 3D structures, highlighting the key residues involved in the recognition process [9,10,11,12].

One notable newly acquired feature of the SARS-CoV-2 S glycoprotein, distinguishing it from so far known CoVs, is the presence of a polybasic four-amino-acid insertion, PRRA, which constitutes a new furin-like cleavage site at the boundary of S1 and S2 subunits [13]; this site is thought to play a role in increased pathogenicity [14]. In fact, the highly pathogenic forms of influenza acquired a furin-like cleavage site cleaved by different cellular proteases, including furin, which are expressed in a wide variety of cell types allowing a widening of the cell tropism of the virus [14,15,16].

A second proteolytic cleavage at site S2′, in the beginning of the S2 subunit, by host cell proteases, typically TMPRSS2, TMPRSS4, or endosomal cathepsins, releases the fusion peptide (FP), which penetrates the host cell membrane, preparing it for fusion. This event triggers the dissociation of S1 subunit and the irreversible refolding of S2 subunit into a post-fusion conformation, a trimeric hairpin structure formed by heptad repeat 1 (HR1) and heptad repeat 2 (HR2) [17]. For these reasons, the identification of drugs targeting either the viral or the host factors could represent a valid strategy to inhibit viral entry [18,19].

Another critical feature of the S protein is its extensive glycosylation, which mediates protein folding and stability and promotes immune evasion by shielding epitopes from neutralizing antibodies [13,20]. Each monomer in the trimeric S protein has 22 N-glycosylation sites, which have been characterized by mass spectrometry experiments [20,21].

SARS-CoV-2 continually accrues genomic mutations as it transmits. A major focus is on variants harboring specific mutations in the S glycoprotein. These mutations may modulate viral properties, such as the mode or rate of transmission, or the ability to cause disease.

One of the first S mutations reported is the D614G. It emerged in late January or early February 2020. Over a period of several months, the D614G mutation replaced the initial SARS-CoV-2 strain identified in China and by June 2020 became the dominant form of the virus circulating globally [22]. Strains harboring this mutation are occurring in more than 96% of all published sequences in Gisaid database, at the date of 13 April 2021. Several studies pointed to the hypothesis that this mutation confers enhanced transmissibility [23], and may increase infectivity by assembling more functional S protein into the virion [24,25]. The cryo-electron microscopy structure of the G614 S variant showed that it adopts a predominantly open conformation, likely promoting a more proficient binding to ACE2, whereas the D614 S is mostly closed [26]. This may account for virus’ observed augmented infectivity and its current predominance.

In December 2020, a novel SARS-CoV-2 variant, namely Variant of Concern 202012/01 (VOC 202012/01, lineage B.1.1.7) emerged and rapidly outcompeted preexisting variants in southeast England [27]. Such variant, also termed the “UK variant”, is defined by 17 mutations, eight of which are in the S glycoprotein (see Figure 1B), and was estimated as having a 43–90% higher reproduction number than preexisting non-VOC variants [28]. In particular, there are three deletions, HV 69–70 and Y144 within the NTD (highlighted in orange in Figure 1 and Figure S2A), and six mutations (N501Y, A570D, P681H, T716I, S982A and D1118H), besides D614G, from which the B.1.1.7 descends. Deletions HV 69–70 in the NTD have been found in multiple independent lineages of SARS-CoV-2 and have been associated with immune escape in immunocompromised patients and enhanced viral infectivity in vitro [29]. Major attention was given to mutations at position 501, as residue N501 within the RBD (green in Figure 1 and Figure S2C) forms important contacts with ACE2 and experimental data suggests that mutations at this site have the potential to increase ACE2 receptor affinity [30]. The N501Y mutation is the most prevalent among all mutations at this position (88% at the date of 5 April 2021), which has occurred since summer 2020 (Figure S1A) and is distributed mainly in Europe, Brazil, and South Africa (Figure S1B). Mutations A570D and D614G are in the S1 subunit between the RBD and the furin-like cleavage site (see Figure 1 and Figure S2B). Mutation P681H, at the S1/S2 boundary, involves the first residue of the canonical furin-like PRRAR↓S cleavage site, newly acquired by SARS-CoV-2 [15], which is important for infection and transmission [14]. Three mutations are in the S2 subunit, i.e., T716I, S982A and D1118H (see Figure 1 and Figure S2A,B).

Other notable variants, which have raised particular concerns, are P.1 from Brazil and B.1.351 from South Africa. The former comprises the two distinct subvariants 28-AM-1 and 28-AM-2, which both carry the K417T, E484K, N501Y mutations, and both developed independently of each other within the same Brazilian Amazonas region [31].

The B.1.351 (also named 20H/501.V2, 20C/501.V2 or 501Y.V2) was first identified in South Africa, where it has rapidly become the predominant strain, raising concerns of increased infectivity and virulence [32]. In fact, mutations in the S protein, which is a key target of developed vaccines and monoclonal antibodies, are of particular interest, as antibody resistance of these variants was reported recently [33,34].

Herein, with the aim to decipher the structural basis of the increased infectivity and virulence reported for the UK variant, we investigate the dynamic profile of full-length SARS-CoV-2 S glycoprotein carrying the eight VOC 202012/01 mutations (henceforth termed UK), in comparison with wild type (henceforth WT), by means of multiple, microsecond-long molecular dynamics (MD) simulations. Both UK and WT S proteins were simulated taking into account the complete glycosylation pattern, consistent with experimental data. 

The MD simulations allowed to capture conformational changes as well as differences in the dynamics between WT and UK systems regarding both the RBD and antigenic sites on the S glycoprotein. This work adds valuable insights on the impact of multiple mutations, carried by the circulating UK variant, on the S protein.

## 2. Results

### 2.1. Altered Flexibility in the UK S Glycoprotein

We investigated the impact of the mutations carried by the UK variants on the structure and dynamics of the S glycoprotein of SARS-CoV-2 by comparing one microsecond MD simulations of WT and UK structures, both in glycosylated form. 

**Figure 2:**
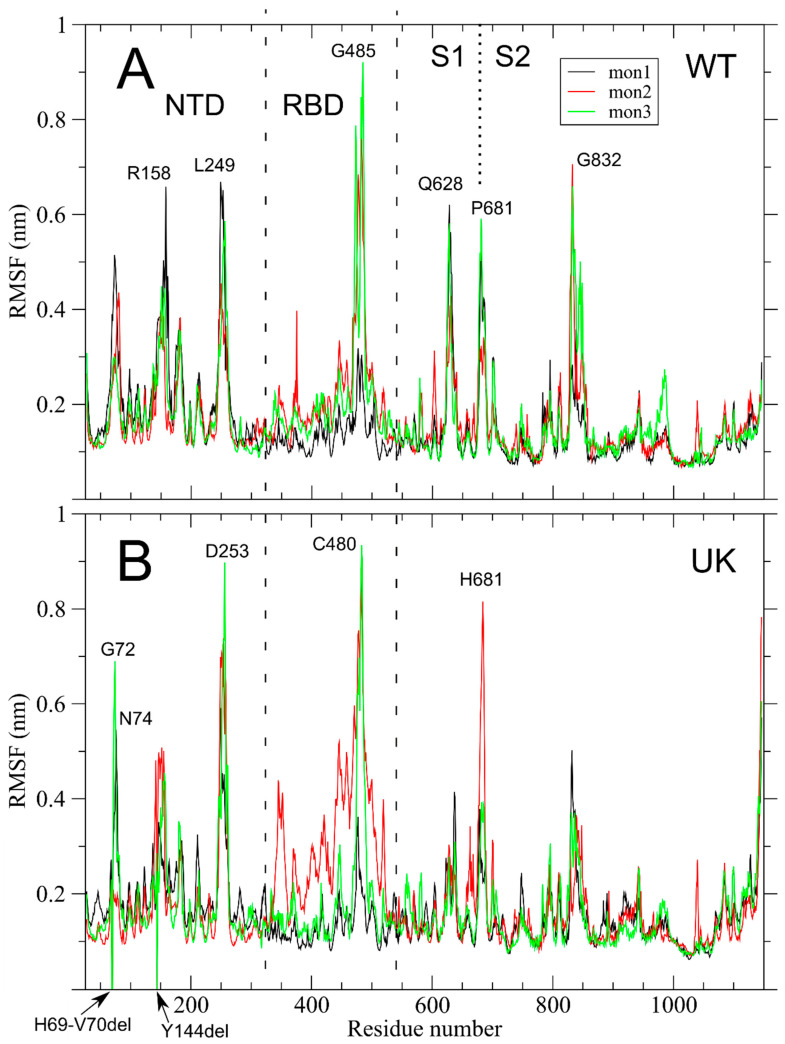
Per-residue RMSF in SARS-CoV-2 WT and UK systems are shown in panels (**A**) and (**B**), respectively. The three S monomers are colored in black, red, and green, respectively. Monomer 2 is in up conformation (see M&M). The NTD and RBD regions are highlighted with dashed lines. The S1/S2 boundary at residue 681 is highlighted with a dotted line. Residue numbers of fluctuation peaks are reported.

Inspection of the per-residue root mean square fluctuations (RMSF), in Figure 2A, reveals a reduced variability among the three monomers and a reduction of RMSF in the NTD in WT as compared to the same system without glycosylation groups (see Figure S3 and Tagliamonte and coauthors [36]). Peak fluctuations are present in the NTD (e.g., R158 and L249) but the highest fluctuations are observed in the RBD, and in particular around residues 480–485 in the RBM. In the same region, the site of mutation N501, which plays an essential role in modulating the interaction with ACE2 [30], does not have a high RMSF value. Other fluctuation peaks in WT are: (i) in the Q628 region, at the C-term of S1 subunit; around P681 that, as already noted, is the first residue of the canonical furin-like PRRAR↓S cleavage sequence; (iii) in the G832 region, mapped to the C-term of the fusion peptide (FP); and (iv) a fluctuation peak in residues 834–847, which is present also in the unglycosylated system (see Figure S3 and Tagliamonte and coauthors [36]). An important structural role of residues 834 to 853, named fusion-peptide proximal region (FPPR) by Cal and coauthors, has been proposed in the transition between up and down conformations of the RBD [35]. 

S mutations in UK structure bring out new fluctuation peaks in NTD (G72 and N74 in monomers 3 and 1, respectively) close to the deletion site 69–70 (see Figure 2B). Note that dummy residues with null fluctuations have been inserted in the RMSF analysis to maintain the residue numbering coherent with WT structure. Other differences in UK structure as compared to WT are: (i) the fluctuation peak around R158 is reduced, although in this case is at residue H146, close to the deletion site 144; (ii) the fluctuation peak in the 249–253 region is increased and in the UK system is nearly as high as the one in the ACE2 binding residues; (iii) the fluctuation peak that is present in WT around residues Q628, is not present in UK; and (iv) the fluctuation peak of residue 681, mutated in histidine, is maintained in UK structure, although only in the up monomer 2, while fluctuations in FPPR are slightly reduced. 

### 2.2. Long Range Correlated Motions in the WT and UK S Glycoproteins

The Essential Dynamics (ED) technique [37] is well suited for the investigation of the long-range protein correlated motions, functional to the different steps in which the S glycoprotein coordinates the host receptor-binding and the proteolytic processing for virus-cell fusion [36]. We applied ED to the concatenated production trajectory of the S monomer (total 2850 ns) and filtered the trajectory along the main eigenvectors (i.e., the ones with higher eigenvalues). Similarly to what observed for the unglycosylated S protein in SARS-CoV-2 and CoVs from bat and pangolin [36], a great portion of the total protein motion are described by the two main eigenvectors V1 and V2 (88.8% in both systems, see Table 1).

**Figure 3:**
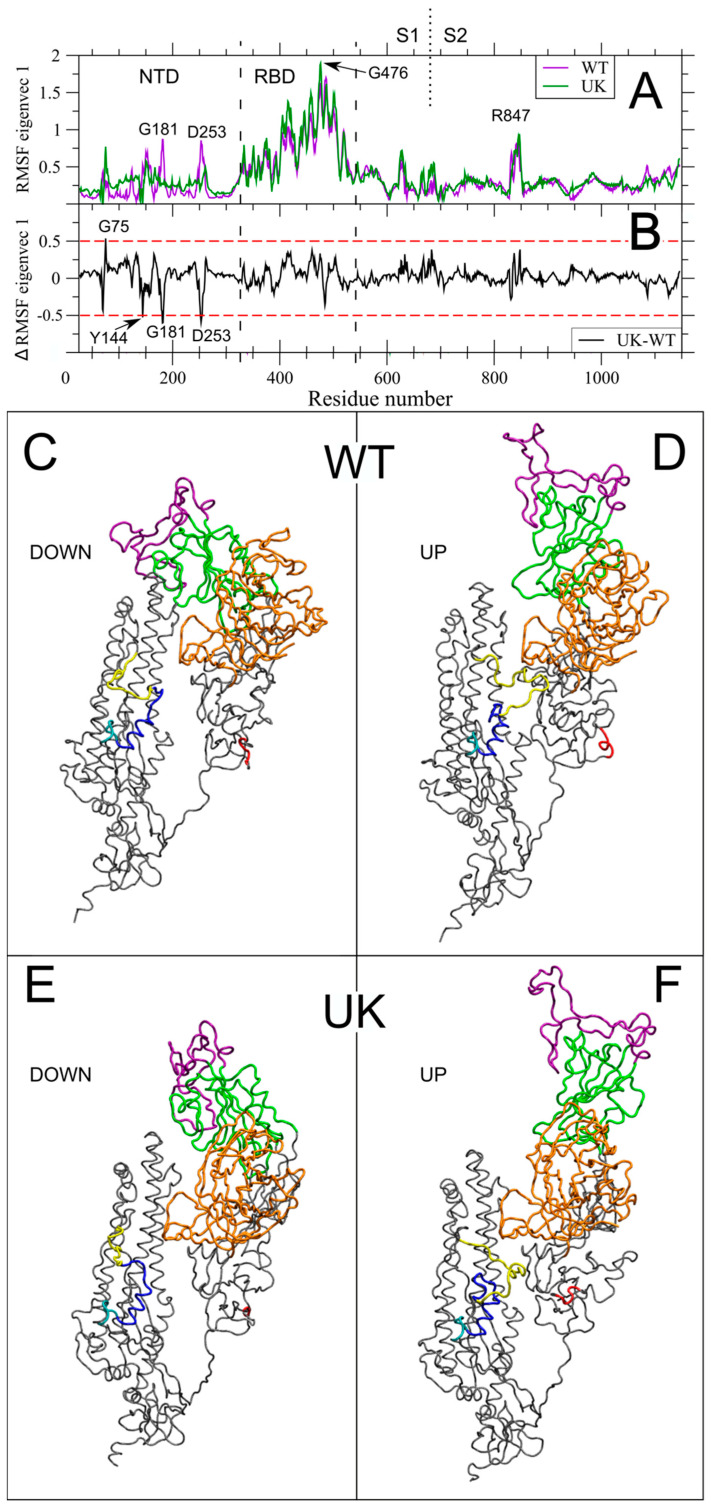
Long-range correlated motions along Essential Dynamics V1. (**A**) RMSF of the S protein filtered trajectory along V1 for WT and UK are reported in purple and dark green, respectively. Residues corresponding to fluctuation peaks along V1 are indicated. (**B**) Difference between filtered RMSF along V1 between UK and WT (black line). Residues above the threshold of |±0.5 nm| (dashed red lines) are indicated. (**C**,**D**) Extreme projection of the S protein MD trajectory along V1 for the WT system. (**E**,**F**) Extreme projection of the S protein MD trajectory along V1 for the UK system. Colors as in Figure 1A. The NTD and RBD regions and the S1/S2 boundary are highlighted as in Figure 2.

In the following, we will discuss the correlated motions described by V1 and V2 in each individual system, that is, we want to highlight the effect of mutations and deletions on the global dynamics on the UK variant. To this end, we have plotted the RMSF along V1 for the WT and UK simulations (see Figure 3A). The figure shows that the motions along V1 are dominated by the rotation of the RBD between down and up conformations, with its peak of fluctuations around G476, and the FPPR region, with its peak around R847. Only two residues show filtered RMSF higher than 0.8 nm outside of these regions (i.e., G181 and D253 in NTD). To highlight this at molecular level, we have plotted the 3D extreme projections along V1 in WT and UK (Figure 3C–F and Supplementary movie 1). An important feature to point out is the similarity of the V1 RMSF between WT and UK, that can be better appreciated in Figure 3B, where the filtered RMSF difference between UK and WT is plotted. In RBD and the whole S2 region, no residues exceed the arbitrary threshold of ±0.5 nm (dashed red line in Figure 3B). A common characteristic in the S protein motion filtered along eigenvector 1 was already observed in different CoVs, ancestor of SARS-CoV-2 [36], suggesting that the observed main motion is indispensable for viral pathogenesis. Moreover, the strongly correlated character of key protein regions such as the RBD and the FPPR, reinforces this hypothesis. However, small differences in V1 between WT and UK are observed in NTD. In particular, the observed two peaks on G181 and D253 are abolished in UK. All the deleted residues have negative RMSF for definition, being their RMSF equal to zero for construction. Only Y144, however, has a value below the −0.5 nm threshold. Interestingly, the absence of H69-V70 in UK is associated with an increase of fluctuation in the close G75. 

**Figure 4:**
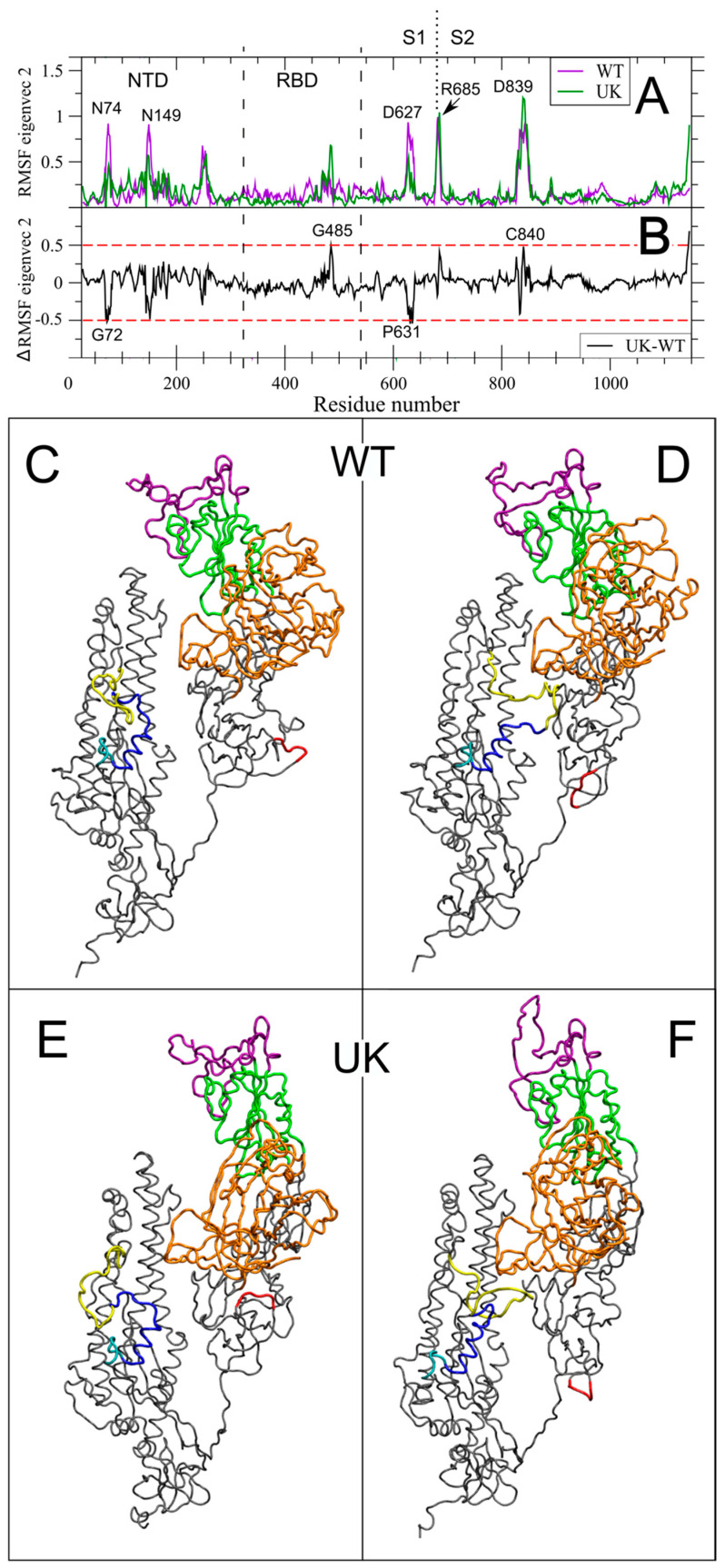
Long-range correlated motions along Essential Dynamics V2. (**A**) Per-residue Root Mean Square Fluctuations (RMSF) in nm of the S protein filtered trajectory along V2 for WT and UK are reported in purple and dark green, respectively. Residues corresponding to peak of fluctuations along V2 are indicated. (**B**) Difference between filtered RMSF along V2 between UK and WT (black line). Residues above the threshold of |±0.5 nm| (dashed red lines) are indicated. (**C**,**D**) Extreme projection of the S protein MD trajectory along V2 for the WT system. (**E**,**F**) Extreme projection of the S protein MD trajectory along V2 for the UK system. Colors as in Figure 1A. The NTD and RBD regions and the S1/S2 boundary are highlighted as in Figure 2.

Similarly to V1, the same analysis has been carried out for V2 in each individual system and the filtered RMSF of the S trajectory along V2 for WT and UK are shown in Figure 4. In line with the dominant role of the RBD rotation in V1, this region does not present motion along V2, particularly in WT (compare Figure 4A with Figure 3A). On the contrary, interesting long range correlated motions in WT involve residues distributed along all the S sequence, i.e., N74 and N149 in NTD, different residues in the D627 region, in the newly acquired furin-like cleavage site (peak in R685, i.e., the bold residue in the PRRAR↓S sequence), and in the FPPR region (peak in D839) that we have shown to have a correlated motion with the up and down RBD rotation along V1. The amplitude of the filtered RMSF along both V2 for these residues is higher than 0.8 nm, and therefore comparable with the peaks in NTD and S2 along V1 for both systems. It is worth noting that V2 accounts for only 17.8% and 15.9% of total variance in WT and UK, respectively, and therefore the occurrence of such an amplitude in the RMSF provides evidence that V2 describes an important long-range correlation movement. An important role of N149, a glycosylated residue, in influencing the RBD up conformation will be discussed in 2.4. This complex network of interactions between distant protein key regions can be appreciated also by looking at the 3D view of the two extreme projections along V2 (Figure 4C,D and Supplementary movies 2,3).

UK variant shows a significant perturbation of these long range correlated motions. As for V1, the differences in the filtered RMSF along V2, between UK and WT, are shown in Figure 4B. Deletion of residues 69–70 determines the abolition of the observed peak of N74, with a variation lower than −0.5 nm in G72. A significant increase in fluctuation is observed in G485, i.e., a residue involved in ACE2 direct contact (purple region in Figure 1) and in in the FPPR region (peak in C840). 

The long range correlated motion captured by V2, therefore, has main characteristics conserved in the UK system, as the peak of RMSF in the newly acquired furin-like region and in the FPPR, close to the peptide fusion region. At the same time, however, the UK system reduces the fluctuations in NTD and in the 627–631 regions, while showing a new correlation peak with the ACE2 binding region, that in WT is limited to the up/down RBD motion along V1. We might speculate that the correlated motion along V2 that connects three S protein key regions is linked to the observed increased infectivity and virulence.

### 2.3. Hydrogen Bond Network in the WT and UK S Glycoproteins

The observed long range protein motions are the consequences of local interactions such as the direct protein–protein hydrogen bonds. We mapped the most stable hydrogen bonds in all the S glycoprotein, i.e., the ones recurring for more than 80% of the MD production time. The number of intra-monomer stable interactions is quite high, with 389, 405, and 420 hydrogen bonds in monomer 1, 2, and 3, respectively. These values are slightly reduced in UK (see Table 2). The number of inter-monomer hydrogen bonds in both systems are higher between the monomers in down conformation, i.e., 1 and 3, while only WT shows a different number of stable hydrogen bonds between M2 and 1 or 3 (31 h-bonds). A heterogeneity role of monomers 1 and 3 is nevertheless observed, due to differences in their hydrogen bond network.

In WT, residues mutated in UK structure are involved in several stable direct hydrogen bonds (gray background in Table S1), with the exception of A570, P681, and T716. The three NTD deleted residues (H69, Y70, and Y144), in particular, are mainly involved in intra-monomer interactions with neighbor residues, yet an intra-monomer interaction between H69 and Y248 is also observed. Notably, the NTD region shows fluctuation peaks in both WT and UK systems (see Figure 2, panel A and B, respectively). N501 is also involved in several stable intra-monomer hydrogen bonds with neighbor residues in RBM. D614, beside an intra-monomer hydrogen bond with A647, shows an inter-monomer interaction Mon1-Mon2 with T859, at the C-term of the FPPR. Interestingly, S982 is involved in three inter-monomer hydrogen bonds with the region 545–547, that link all the three monomers among themselves. Moreover, S982 in monomer 2 (the one in up position) has two stable intra-monomer interactions with neighbor residues. D1118 is also highly involved in four intra-monomer interactions in monomer 2 and one in monomer 1 with R1091 and T1116. Interestingly, an inter-monomer interaction between D1118 in monomer 2 and R1091 in monomer 1 is also observed. Indeed, D1118 and R1091 of all three monomers interact during the simulations even if not all the interactions are reported in Table S1 because, for example, the switch of the acceptor atom between OD1 and OD2 in D1118 of monomer 3, brings the single interaction under the chosen cut-off of 80% of simulation time. 

The complex hydrogen bond network of these residues in WT is naturally perturbed by their mutation in UK variant. The stable hydrogen bonds observed in UK are reported in Supplementary Table S2 (again the mutated residues are indicated by a gray background). Besides the loss of the interactions by the deleted residues in NTD, Y501 shows only two intra-monomer stable interactions, with Q498 in monomer 2 and Y449 in monomer 3. While D570 does not show stable interactions in WT, in UK structure it forms both intra-monomer interactions with neighbor residues than inter-monomer ones with S967 and K854, connecting monomer 2 with 1 and 3, respectively. The residue G614, as expected, is not involved in interactions, while H681 and I716, two residues that have no stable interactions in WT, show four and one intra-monomer bonds with neighboring residues, respectively. On the contrary, A982 does not show stable interactions, thus losing the inter-domain interactions observed previously among all the monomers in WT. Residue 1118, mutated in histidine, maintains the strong interaction with R1091 through an intra-monomer bond in monomer 1 and three inter-monomer bonds. Interestingly, an inter-monomer bond between two H1118 is also observed, between monomer 1 and 2. Additionally, the rich hydrogen bond network of H1118 comprises an intra-monomer bond with T1116. 

**Figure 5:**
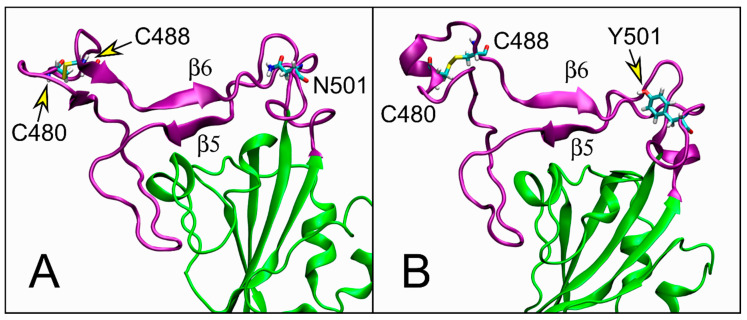
Cartoon representation in the RBM region. (**A**) WT snapshot after 629.8 ns of MD simulation, centroid of the most populated protein cluster. Colors as in Figure 1D,E. Side chains of residues 480, 488, and 501 are shown in licorice view. (**B**) UK snapshot after 840.5 ns of MD simulation, centroid of the most populated protein cluster.

The analysis of hydrogen bonds also reveals how the conservative mutation N501Y does not strongly perturb the RBM fold responsible for receptor binding. The close β-sheet formed by β5 (residues 454–455) and β6 (residues 492–495; numbering as in Lan and coauthors [11]), for example, remains stable in the UK system (see Figure 5), while subtle perturbation appears in the region of other two ACE2 binding residues, i.e., Q474, F486 [10]. In the simulation, that region in WT shows propensity to form a β-sheet formed by two β-strands in residues 473–475 and 486–490. Note that in the second β-strand C488 that forms a disulfide bond with C480 is included (Figure 5A). In UK we observe a conformational switch with the loss of the β-sheet and a propensity for an α-helix in residues 479–484 (see Figure 5B). This conformational switch can explain the fluctuation peak in C480 in UK (Figure 2B) and the correlated motion in the region G485, again only in UK, along ED eigenvector 2 (Figure 4B).

### 2.4. N-Glycans Modulate the RBD Dynamics in the WT and UK S Glycoproteins

An important feature of SARS-CoV-2 S protein is its extensive glycosylation, comprising 18 N-glycosylation sequons [20] per monomer and two O-glycosylation [21] sites. Glycans, beyond shielding of epitopes from antibody recognition, play a key role on structure and dynamics of S proteins. In particular the glycans at N165 and N234 stabilize the RBD “up” conformation as evidenced by multi-microsecond MD simulations and biolayer interferometry experiments [38].

We investigated the effect of mutations in UK S glycoprotein, specifically the H69, Y70 and Y144 deletion, on the interaction, mainly due to hydrogen bonds, between RBD and glycans that modulate the dynamics and the mechanism of opening of RBD domain. The stability of the hydrogen bonds was evaluated by the calculation of the occupancy, defined as the fraction of MD time in which the hydrogen is formed with respect to the MD production time.

**Figure 6:**
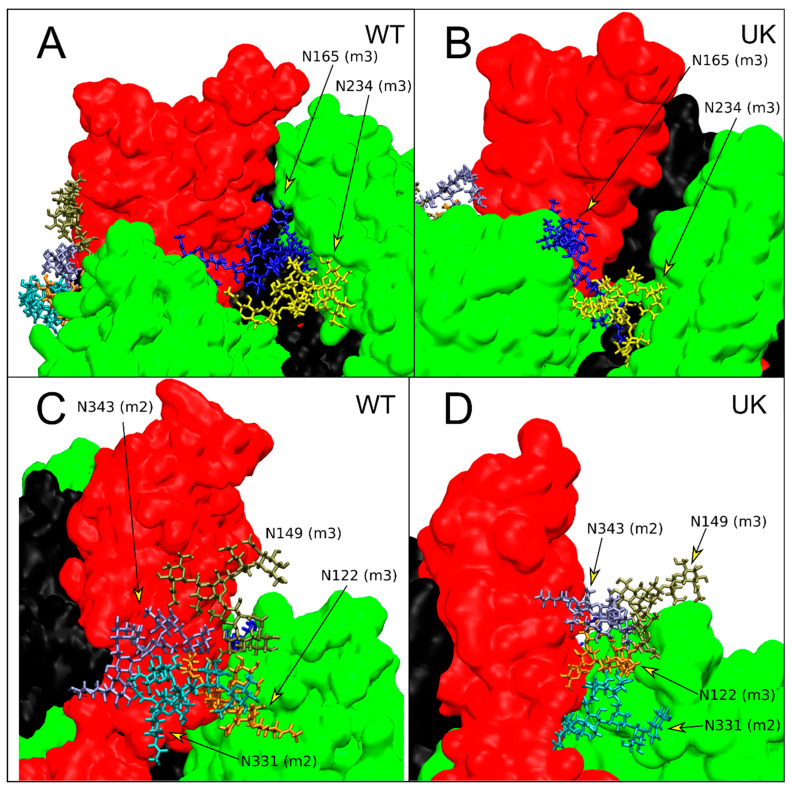
Interactions of N-glycans with RBD domain in up conformation (monomer 2, red color). Monomer colors as in Figure 1C. (**A**,**B**) Enlargement of the region of interaction of N165 and N234 of monomer 3 (green color) for WT and UK, respectively. (**C**,**D**) Enlargement of the region of interaction of N122 and N149 of monomer 3; N331 and N343 of monomer 2 (red color) for WT and UK, respectively. For each residue, the monomer to which they belong is reported in parentheses.

In WT the glycan at N165 (biantennary FA2G2S1) of the monomer 3 penetrates into the cavity created by the opening of RBD of the monomer 2, forming a dynamic network of hydrogen bond with the amino acids of the RBD portion of monomer 2 (Figure 6A). Specifically, the glycan at N165 makes stable hydrogen bonds with E516 (82% occupancy) and R357 (35% occupancy) and interacts with N394, K462, and H519 with an occupancy greater than 15% (Table S3). The longer antenna, with the sialic acid at the end, of the glycan at N165 makes hydrogen bonds also with some amino acids of the RBD of monomer 3 (intra-monomer interaction) mainly with Y369, N370, and S373 for more than 20% of the MD production time.

In the case of glycan linked to N234 of the monomer 3 (M9) we do not observe a direct stabilization of the up conformation of RBD (see Figure 6A). In fact, the glycan at N234 makes stable hydrogen bonds with the amino acids of RBD of monomer 3 (intra-monomer interaction) in particular with D389 (86% occupancy), N388 (90% occupancy), Y369 (72% occupancy), and T385 (72% occupancy) and interacts stably with the glycan at N165 for more than 83% of MD production time (Table S4). 

In WT S glycoprotein we observed other interesting interactions between RBD in up conformation (monomer 2) and the glycans at N122 (M5) and N149 (FA2) of monomer 3 (Figure 6C). These two glycans make stable hydrogen bonds with the amino acids of the RBD and with the glycan at N331 (FA2) and N343 (FA1) of monomer 2. Specifically, the glycan at N122 makes hydrogen bonds with the amino acids of RBD (Table S5) with an occupancy of 55% and with the glycan at N331 and N343 with an occupancy of 95% and 50%, respectively (Table S4). The glycan at N149 interacts mainly with the amino acids of RBD in up conformation with an occupancy of 94% (Table S5) and with the glycan at N343 of monomer 2 with an occupancy of 32% (Table S4). The glycan–glycan interaction between N149 of monomer 3 and N331 of monomer 2 shows an occupancy lower than 2% of the production time. 

In the case of UK mutant, the deleted residues in the NTD region (H69, Y70, and Y144) affect not only the structure and dynamics of protein but also the orientation of the N-glycans in this domain. 

As shown for the UK mutant, the glycan linked to N165 of monomer 3 shows a different orientation with respect to WT (see Figure 6B) that significantly reduces the interaction with the RBD in up conformation (15% occupancy, Table S5). Only the amino acids R357 and N396 of monomer 2 are involved in the hydrogen bond interaction (Table S6). In UK, one antenna of glycan at N165 is oriented towards the water phase while the other antenna (with the sialic acid at the end) is oriented towards the RBD of monomer 3 (Figure 6B) and stably interacts, through hydrogen bonds, with the glycan at N234 (97% occupancy, Table S4). The glycan at N234 in UK mutant, as in WT, interacts exclusively with the amino acid of RBD of monomer 3 (Table S6). As for the glycans at N122 and N149, the effect of deletions H69, Y70, and Y144 profoundly influences their orientation and, as a consequence, their ability to interact with RBD in up conformation with respect to WT. Specifically, only the glycan linked to N122 makes hydrogen bonds with amino acids of RBD in up conformation with a significantly lower frequency with respect to WT. 

The three NTD deleted residues in the UK mutant influence also the glycan–glycan interaction between the glycan at N122 and N149 of monomer 3 and the glycan linked to N331 and N343 of the monomer 2 (Figure 6D). In UK mutant, the glycan at N343 makes more stable hydrogen bonds with both the glycans at N122 and N149 of monomer 3 with respect to WT with an occupancy of about 97% (Table S4). On the contrary, the glycan at N331 of monomer 3 interacts only with the glycan at N122 with an occupancy of 87%, slightly lower with respect to WT (95% occupancy). 

## 3. Discussion

In the present study, we have pointed out key characteristics of the dynamics of S glycoprotein in the WT and mutant form identified as UK. MD revealed substantially different motions in the UK mutant with respect to the WT, also considering the influence of glycosylation on the fluctuations of the protein domains. Regarding the UK S glycoprotein, the analysis of RMSF revealed that deletions sites at positions 69–70 and 144 within the NTD induce substantial fluctuations in the surrounding residues (Figure 2). Both WT and UK S glycoproteins show similar motions of residues involved in ACE2 binding. The higher mobility of residue 681 was consistently observed in the three WT monomers, whereas in the UK S only the up monomer 2 displayed higher fluctuations. This result is in line with previous studies in which an allosteric effect on RBD positioning was triggered by changes in the region surrounding the furin cleavage site [39]. Therefore, the acquisition of the D614G and P681H mutations might have resulted in a S glycoprotein more prone to protease cleavage, even though it is still far from clear if the P681H mutation itself may confer an advantage with regard to transmissibility [40]. 

Then, we extracted the correlated motions of the WT and UK S monomers using essential dynamics analysis. The two main eigenvectors V1 and V2, with the largest associated eigenvalues, account for the maximum protein dynamics, with the rotation of RBD being dominant in V1 (Figure 3). Interestingly, the long range correlated motion between the 834–853 FPPR region, which has a crucial role in membrane fusion, and the up and down RBD rotation along ED V1 is present in both WT and UK (Figure 3), and observed also in SARS-CoV-2, Bat-CoV and Pangolin-CoV-2017 unglycosylated forms [36]. On the other hand, long-range correlated motions among distant regions of the S glycoprotein, including residues involved in ACE2 binding, the newly acquired furin-like PRRAR↓S cleavage site, and peptide fusion region, emerged for the UK system along V2 (Figure 4). Intriguingly, this behavior might account for the observed increased infectivity and virulence of the UK mutant.

An examination of the hydrogen bond network (Table 1, Tables S1 and S2) indicated interesting changes in hydrogen bonding interaction patterns in the UK S protein in comparison with the WT. The monomers in down conformation showed a higher number of inter-monomer hydrogen bonds with respect to the monomers up in both WT and UK systems. In the UK system, the three deletions within the NTD induce a perturbation of the inter-domain contacts, which could influence this crucial antigenic region, eventually leading to the observed resistance to antibodies targeting the NTD [33,41]. 

The most striking result is the switch of the β-sheet to an α-helix in residues 479–484 in the UK system (Figure 4), which highlights the inherent higher plasticity of the RBM. 

Interestingly, mutations at residue E484 (such as E484K found in P.1 from Brazil) have been demonstrated to reduce neutralization by both monoclonal antibodies and human sera or plasma [42,43,44], suggesting that this residue functions as an antigenic “hotspot”. Thus, it seems that this area is subjected to a certain selective pressure favoring viral escape.

On the other hand, mutation at N501 alone does not strongly perturb the RBM fold responsible for receptor binding; however, the combination of N501Y with other mutations (such as K417N and E484K) may cause a larger decrease in neutralization [45,46].

It is known that glycosylation has crucial roles in viral pathobiology, such as protein proper folding and viral tropism [20]. Glycosylation modulates accessibility to host proteases and facilitates immune evasion by shielding epitopes from neutralizing antibodies [20]. Recent work carried out by Casalino et al. evidenced an essential structural role of glycans in modulating the conformational transitions of the RBD, with the “up” and “down” conformations of RBD showing a remarkably different extent of glycan shield [38]. A recent study reported that glycan decoration might even be more extensive on S glycoproteins assembled in infected cells than on S expressed recombinantly [47]. In this work, the analysis of glycan–protein and glycan–glycan hydrogen bonds between the RBD of monomer 2 and the NTD of monomer 3 reveals that glycans contribute to modulate the flexibility of RBD and regulate the dynamics of the opening state of the RBD domain. Therefore, the differences observed in the simulation on the glycan–protein and glycan–glycan hydrogen bond network in WT and UK can explain the different flexibility of the RBD domain in these two proteins. In WT the interaction of the glycan at N165, N122 and N149 with the amino acids of RBD and the glycan–glycan interactions stabilize the up conformation of the RBD. In UK the RBD domain in up conformation interacts with the NDT of the monomer 3 exclusively by hydrogen bonds between glycans. Due to the fast dynamics of glycans, the RBD in up conformation of UK shows higher flexibility with respect to WT. This can explain the major fluctuation of the RBD in UK mutant with respect to WT protein observed in the per-residue RMSF (Figure 2) and the network of long-range correlation movements connecting the RBM, residues at NTD, the newly acquired furin-like region and the FPPR, close to the peptide fusion region along ED eigenvectors 1 and 2 (Figure 3 and Figure 4). Therefore, in WT, the up conformation of the RBD is stabilized by a dense network of interactions between the glycans and the RBD residues, as well as glycan–glycan interactions. In the UK, the RBD up interacts with the NDT of the monomer 3 exclusively by glycan–glycan interactions. Thus, the RBD up in the UK system experiences higher fluctuations with respect to WT. This behavior might be responsible of the higher proportion of RBD up state particles observed in UK mutant Cryo-EM structures, due to a possible reduction of the transition barrier to the up state [48].

The UK variant, as well as other VOCs, pose a threat for antibodies and vaccines development; in this regard, interesting approaches are being investigated to overcome the reported resistance to mutant S, such as antibodies targeting multiple epitopes on the viral S [41], broadly neutralizing antibodies targeting conserved epitopes among Sarbecoviruses [49], as well as multiplexed-chimeric spike vaccines, to increase coverage against CoVs infections [50].

## 4. Materials and Methods 

### 4.1. Modeling of S Glycoprotein Starting Structures

The model of 3D structure of prefusion form of the S glycoprotein in the open conformation was built by using SWISS MODEL [51] by alignment of the reference sequence (NCBI YP_009724390.1, UniProt: P0DTC2 SPIKE_SARS2) to the cryo-EM structure (PDB ID: 6VYB) [13]. It was necessary, in fact, to restore the furin cleavage site sequence to the original SARS-CoV-2 one (Wuhan-Hu-1 strain), that is mutated in the cryo-EM experimental structure (henceforth WT). Loops and missing side chain were built automatically by SWISS MODEL. Note that the starting cryo-EM structure has a single S monomer in “open” conformation, i.e., in which the monomer identified with chain B has the RBD in a conformation favorable to interact with the human ACE2 receptor (“up”).

### 4.2. UK Mutant

The structure of mutant protein was built by changing the reference sequence and using the same cryo-EM structure (PDB ID: 6VYB) as template in SWISS MODEL. In particular, we modified the WT 3D structure to model the UK variant identified in the S glycoprotein Lineage B.1.1.7, i.e., 69–70 HV del, Y144 del, N501Y, D614G, P681H, T716I, S982A, and D1118H. 

### 4.3. Glycosilation of S Protein

The resulting protein structure of spike glycoprotein WT and UK mutant (residues 27–1147) contains 18 N-glycosylation sites and two O-glycosylation sites per monomer [20,21], detailed in Table S7. The glycosylation of N- and O- sites was built by using the glycoprotein builder available at GLYCAM-Web (www.glycam.org, accessed on 13 April 2021). An asymmetric glycosylation of the three monomers of each protein has been performed and the structure of glycans has been derived by glycoanalytic data for the N-glycans [20] and O-glycans [21] and according to the work of Casalino et al. [38]. It is worth noting that the model of Casalino et al. of the S protein (residues 16–1234) includes the “head” region (residues 16–1140), with 19 sites of glycosylation, the “stalk” region (1141–1234), with three sites of glycosylation and the transmembrane region (1235–1274). To build our model, based on the 6VYB structure (residues 27–1147), only the 18 sites of glycosylation that are present in this region of the protein were considered.

Steric clashes between glycans and amino acid residues were removed by a manipulation of glycan dihedrals or asparagine side chain involved in the N-glycosidic bond.

### 4.4. Molecular Dynamics Simulation and Analyses

The proteins were modeled using Amber14SB force field [52] and the GLYCAM06 force field [53] was used for the carbohydrate moieties. The AMBER topology files of glycosylated proteins, obtained by GLYCAM-web server, were converted into the GROMACS format using the ACPYPE script [54]. Titratable residues of proteins were modelled according to their protonation state in water at pH = 7. Proteins were embedded in a triclinic box, extending up to 15 Å from the solute, and immersed in TIP3P water molecules [55]. Counter ions were added to neutralize the overall charge with the genion GROMACS tool. Short-range interactions were calculated using a cut-off of 1.0 nm and the long-range electrostatic interactions were accounted by using the particle mesh Ewald method (PME) [56] with a grid spacing of 0.12 nm and a spline interpolation of order 4. All h-bonds were constrained using the P-LINCS algorithm [57,58] whereas the geometry of water molecules was fixed with the SETTLE algorithm [59]. After energy minimizations, the systems were slowly relaxed for 5 ns by applying positional restraints of 1000 kJ mol^−1^ nm^−2^ to the protein atoms. Following this step, unrestrained MD simulations were carried out for a length of 1000 ns for each system, with a time step of 2 fs, using GROMACS 2020.2 simulation package [60]. All the simulations were performed on the supercomputer Marconi-100, CINECA, Bologna, Italy, as in previous work [61]. V-rescale temperature coupling was employed to keep the temperature constant at 300 K [62] and the Parrinello–Ramhan barostat [63] was used to maintain constant the pressure at 1 bar. The first 50 ns of each trajectory were excluded from the analysis.

Per-residue Root Mean Square Fluctuations (RMSF) were calculated with the GROMACS rmsf tool. Dummy residues with null fluctuations were inserted in the RMSF analysis of UK mutant to maintain residue numbering coherent with WT. The Essential Dynamics (ED) analysis was carried out to identify the main 3D directions along which the majority of the protein motion is defined [37]. ED analysis is based on the diagonalization of the covariance matrix built from the atomic fluctuations after the removal of the translational and rotational movement [37]. It is usually applied only on the c-alpha atoms since they describe the motion of the protein main chain [37]. ED analysis was carried out using the GROMACS covar and anaeig tools on the concatenated trimeric trajectory. Main principal component movements were checked to be conserved in different time windows. 

A cluster analysis has been carried out on the WT and UK trajectories using the GROMACS cluster tool, according to the algorithm described by Daura and coauthors [64]. Briefly, the algorithm counts the number of neighbors using a cut-off of 0.1 nm, then takes the structure with the largest number of neighbors with all its neighbors as a cluster and eliminates it from the pool of clusters. The process is then repeated for the remaining structures in the pool. For both systems, a total of 9000 frames were analyzed and clustered. For WT, the first cluster contains 3592 frames and the centroid (i.e., the frame closer to the middle structure) corresponds to a simulation time of 629,800 ps. For UK, the first cluster contains 3506 frames, and the centroid corresponds to a simulation time of 840,500 ps.

Hydrogen bonds analysis was performed by using GROMACS hbond tool and VMD [65] in combination with in-house Tcl scripts. Hydrogen bonds were identified by using the geometric criteria; we considered two functional groups linked by hydrogen bond when the distance between heavy atoms (Donor–Acceptor) was less than 0.35 nm and the angle Acceptor–Donor–Hydrogen was smaller than 30°. Occupancy (%) was obtained by counting the time in which a hydrogen bond was formed with respect to the total MD production time.

## 5. Conclusions

This work presents MD simulations of the full-length model of the WT and UK mutant SARS-CoV-2 S glycoprotein. The analyses, carried out on a microsecond time scale, showed the high level of complexity of the S glycoprotein dynamics. The mutations in the UK form established a sophisticated balance between the motions of distant regions, highlighting an interesting crosstalk between the RBD and the S2 subunit. Additionally, protein–glycan and glycan–glycan analyses outline a different network of interactions between the WT and UK forms, possibly resulting in different shielding and also influencing the RBD fluctuations.

Overall, this work brings new insights on this critical molecular target and points to opportunities and challenges for antibodies and vaccine design. In the future, it will be of pivotal importance continuing to track VOCs and VOIs (variant of interest), and to monitor how the acquired mutations affect the epitopes on the S glycoprotein that are targeted by the vaccines and antibodies against SARS-CoV-2. The integration between computer simulations and experimental work opens the possibility of synergies that could be of great help in fighting the current global pandemic.

## Figures and Tables

**Table 1:** Percentage of global motion described by ED eigenvectors 1–3 (named V1, V2, and V3, respectively) in WT and UK.

	V1	V2	V3
**WT**	71.0%	17.8%	1.6%
**UK**	72.9%	15.9%	1.8%

**Table 2:** Number of direct protein–protein hydrogen bonds recurring for more than 80% of simulation time in Monomer 1, 2, and 3. Intra-monomer interactions are reported in the first three columns, while the inter-monomer ones are in the last three columns.

	M1-1	M2-2	M3-3	M1-2	M1-3	M2-3
**WT**	389	405	420	24	41	39
**UK**	376	400	415	31	42	31

## Data Availability

Not applicable.

## Supplementary Materials

Supplementary materials can be found at https://www.mdpi.com/article/10.3390/ijms22115464/s1.

## Abbreviations

COVID-19	Coronavirus disease 2019
SARS-CoV-2	Severe acute respiratory syndrome coronavirus 2
CoVs	Coronaviruses
NTD	N-terminal domain
RBD	Receptor binding domain
RBM	Receptor binding motif
ACE2	Angiotensin-converting enzyme 2
FP	Fusion peptide
HR1	Heptad repeat 1
HR2	Heptad repeat 2
VOC	Variant of concern
VOI	Variant of interest
WT	Wild-type
MD	Molecular dynamics
RMSF	Root mean square fluctuations
ED	Essential dynamics
