# Supplementary material for: Altered Local Interactions and Long-Range Communications in UK Variant (B.1.1.7) Spike Glycoprotein
Source: Int J Mol Sci. 2021 May 22;22(11):5464. doi: 10.3390/ijms22115464 (PMC8196891; doi:10.3390/ijms22115464)
Supplement: Supplementary file 1 [file ijms-22-05464-s001.zip › Supplementary_Materials/Movies_Legend.pdf]

Supplementary movies

Movie 1. Comparison of essential dynamics motion along eigenvector 1 for WT and UK. Domain colors as in in Figure 1A. The most correlated motions involve the RBD and the FPPR (see Figure 3).

Movies 2-3. Essential dynamics motion along eigenvector 2 for WT and UK, respectively. Correlated motions along this eigenvector highlight a network of interactions between distant protein key regions in both systems (see Figure 4).
