# Supplementary material for: Altered Local Interactions and Long-Range Communications in UK Variant (B.1.1.7) Spike Glycoprotein
Source: Int J Mol Sci. 2021 May 22;22(11):5464. doi: 10.3390/ijms22115464 (PMC8196891; doi:10.3390/ijms22115464)
Supplement: Supplementary file 1 [file ijms-22-05464-s001.zip › Supplementary_Materials/Supplementary Materials.pdf]

Supplementary Materials

Altered Local Interactions and Long-range Communications in UK Variant (B.1.1.7) Spike Glycoprotein

Stefano Borocci[1,2], Carmen Cerchia[3], Alessandro Grottesi[4], Nico Sanna[1], Ingrid Guarnetti Prandi[5], Nabil Abid[6,7], Andrea R. Beccari[9], Giovanni Chillemi[1,8,*], Carmine Talarico [9,*]

1 Department for Innovation in Biological, Agro-food and Forest systems, DIBAF, University of Tuscia, via S. Camillo de Lellis s.n.c., 01100 Viterbo, Italy; borocci@unitus.it (S.B.); n.sanna@unitus.it (N.S.); gchillemi@unitus.it (G.C.)
2 Institute for Biological Systems, ISB, CNR, Via Salaria, Km 29.500, 00015 Monterotondo, Rome 00015, Italy
3 Department of Pharmacy, University of Napoli "Federico II", via D. Montesano 49, 80131 Napoli, Italy; carmen.cerchia@unina.it (C.C.)
4 Department HPC, CINECA, via dei Tizii 6, 00185 Roma, Italy; a.grottesi@cineca.it (A.G.)
5 Department of Chemistry and Industrial Chemistry, University of Pisa, via Giuseppe Moruzzi 3, I-56124 Pisa, Italy; ingrid.prandi@gmail.com (I.G.P.)
6 Laboratory of Transmissible Diseases and Biological Active Substances LR99ES27, Faculty of Pharmacy, University of Monastir, Rue Ibn Sina, 5000, Monastir, Tunisia; nabilabidbensalem.2014@yahoo.fr (N.A)
7 High Institute of Biotechnology of Sidi Thabet, Department of Biotechnology, University of Manouba, BP-66, 2020, Ariana-Tunis, Tunisia
8 Institute of Biomembranes, Bioenergetics and Molecular Biotechnologies, IBIOM, CNR, Via Giovanni Amendola, 122/O, 70126, Bari, Italy
9 Dompé Farmaceutici SpA, via Campo di Pile, 67100 L'Aquila, Italy; carmine.talarico@dompe.com (C.T.); andrea.beccari@dompe.com (A.R.B.)
* Correspondence: gchillemi@unitus.it (G.C.); Carmine.Talarico@dompe.com (C.T.)

Figure S1. Worldwide distribution of N501Y mutation (7432 genomes sampled between Dec 12, 2019 and Mar 5, 2021) predicted using Nextclade.

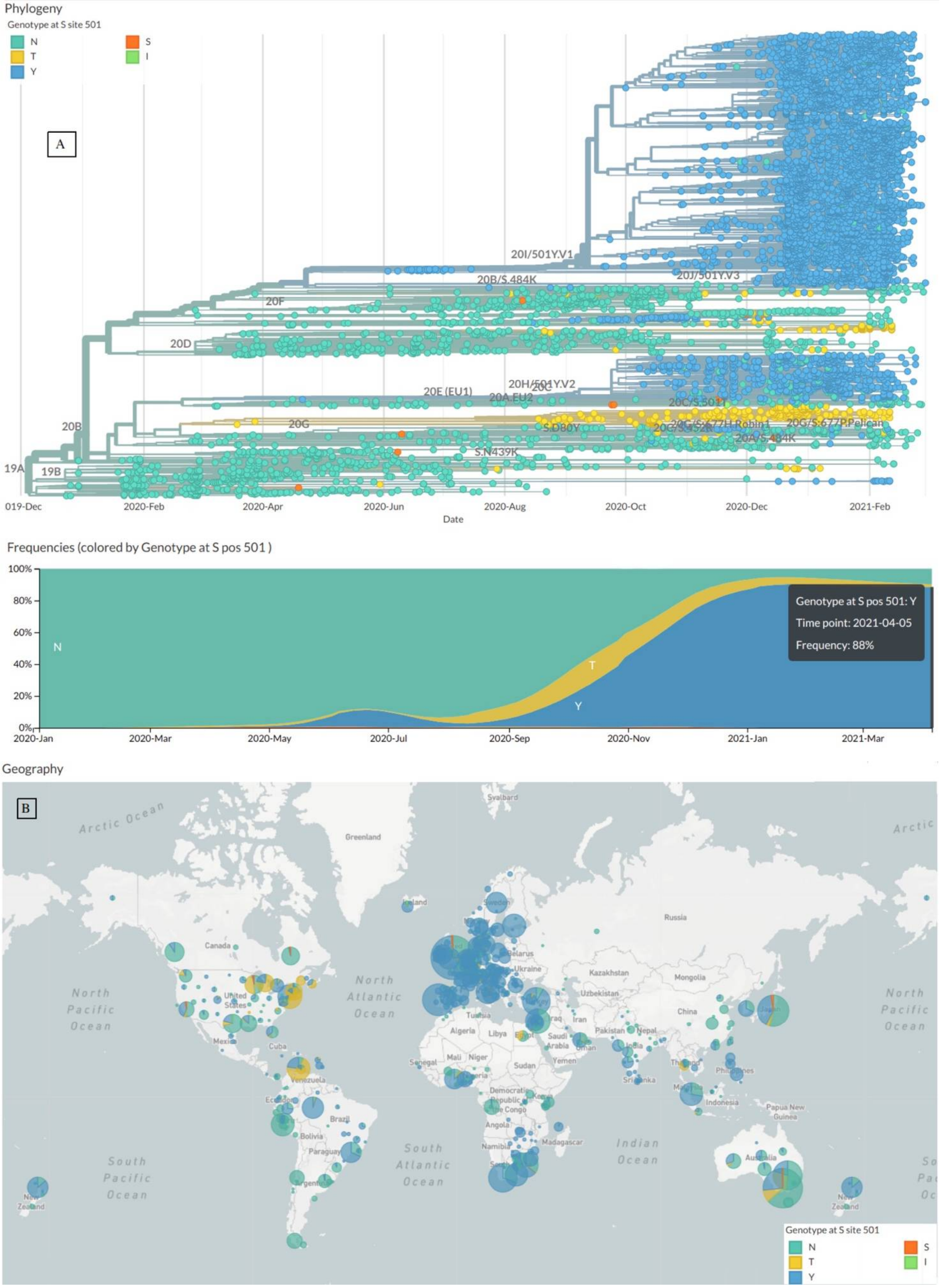

Figure S2. The site of mutations in VOC 202012/01 S protein (see Figure 1B) are shown as licorice in chain B (up conformation) of the WT (MD snapshot after 629.8 ns of MD simulation, centroid of the most populated protein cluster). A) The three residues deleted in VOC 202012/01 are in NTD (orange). S982 is one of the three mutation sites in the S2 region. B) A570 and D614 are in the region between RBD and the furin-like cleavage site. P681 is the first residue of the PRRAR cleavage sequence. The other two mutation sites in S2, T716 and D1118, are also shown. C) N501 is the only mutation located in RBD, particularly in RBM.

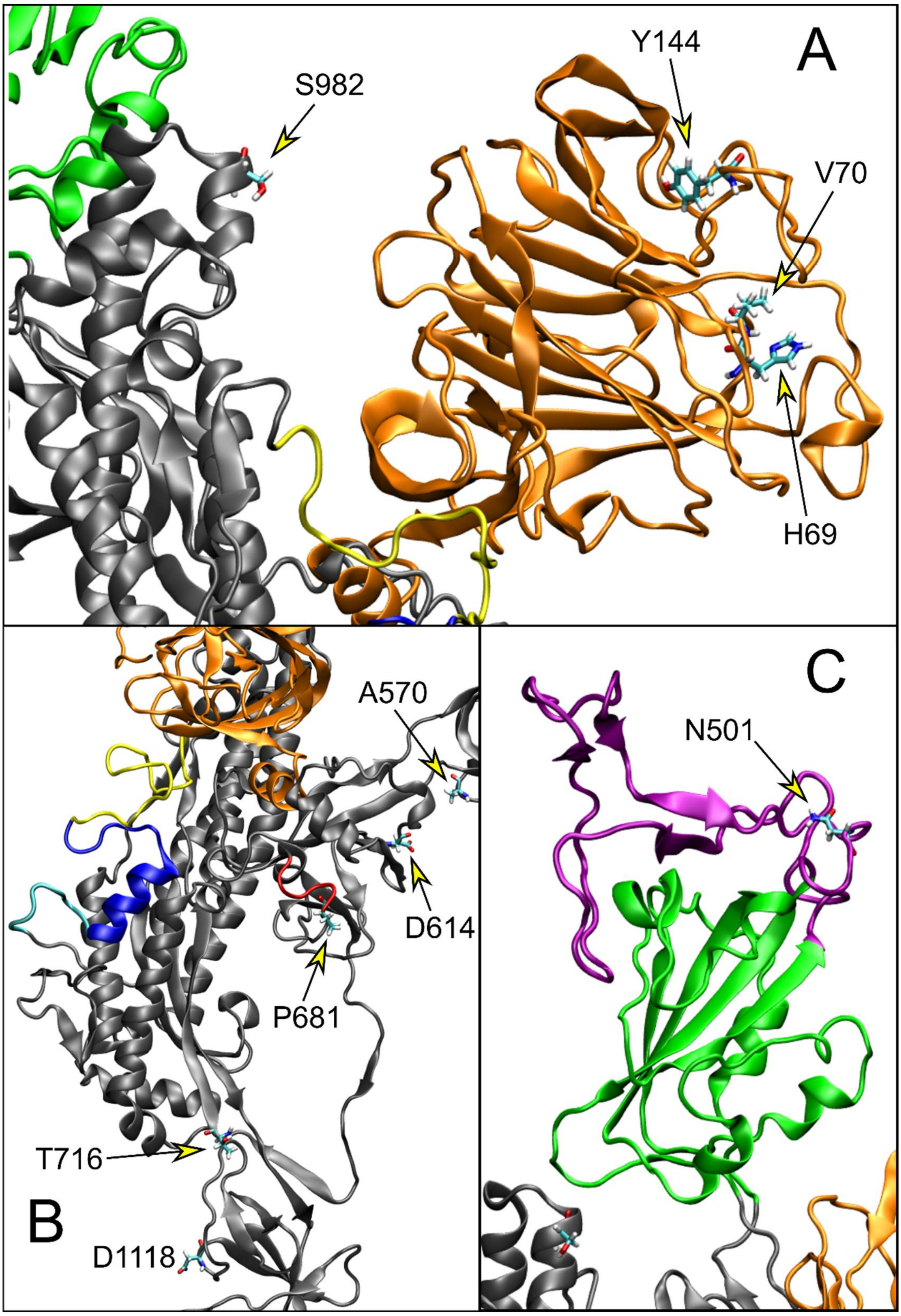

Figure S3. Per-residue RMSF in SARS-CoV-2 WT unglycosylated S protein MD simulation, as described in Tagliamonte and coauthors [1]. In this case, monomer 1 (blue line) is in up conformation.

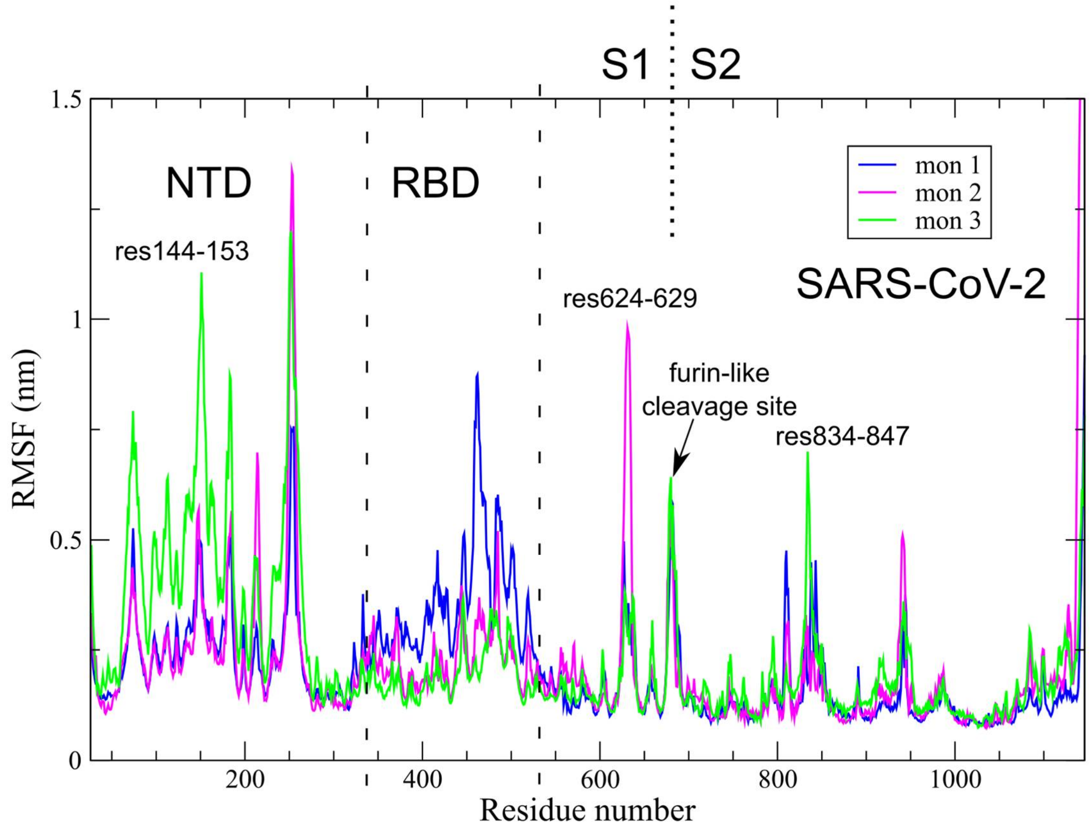

Table S1. Direct protein-protein hydrogen bonds recurring for more than 80% of simulation time involving residues site of UK mutations (gray background) in WT S glycoprotein.

Donor Residue	Donor atom	Donor Monomer	Acceptor residue	Acceptor atom	Acceptor Monomer	% residence time
GLY72	N	1	HIS69	O	1	100.0
TYR248	OH	1	HIS69	ND1	1	95.8
SER71	N	2	HIS69	ND1	2	87.5
GLY72	N	2	HIS69	O	2	91.7
SER71	OG	3	HIS69	ND1	3	94.8
GLY72	N	1	VAL70	O	1	95.8
TYR144	N	2	GLY142	O	2	100.0
TYR144	OH	2	ASN121	O	2	93.8
TYR144	OH	2	VAL159	O	2	93.8
GLN498	NE2	2	ASN501	ND2	2	90.6
ASN501	ND2	2	GLN498	OE1	2	95.8
GLN506	NE2	2	ASN501	O	2	81.3
GLY502	N	3	ASN501	OD1	3	97.9
GLN506	NE2	3	ASN501	O	3	100.0
ASP614	N	1	ALA647	O	1	96.9
THR859	OG1	2	ASP614	OD1	1	87.5
SER982	OG	1	THR547	OG1	3	84.4
THR547	OG1	2	SER982	OG	3	95.8
SER982	N	2	ASP979	O	2	87.5
SER982	OG	2	GLY545	O	1	88.5
SER982	OG	2	ASN978	O	2	99.0
ARG1091	NH1	1	ASP1118	O	1	99.0
ARG1091	NH1	1	ASP1118	OD2	2	100.0
ARG1091	NH1	2	ASP1118	OD1	2	99.0
ARG1091	NH1	2	ASP1118	O	2	97.9
THR1116	OG1	2	ASP1118	OD1	2	91.7
THR1116	OG1	2	ASP1118	OD2	2	90.6

Table S2. Direct protein-protein hydrogen bonds recurring for more than 80% of simulation time involving mutated residues in UK S glycoprotein.

Donor Residue	Donor atom	Donor Monomer	Acceptor residue	Acceptor atom	Acceptor Monomer	% residence time
GLN498	NE2	2	TYR501	OH	2	92.7
TYR449	OH	3	TYR501	OH	3	99.0
THR572	N	1	ASP570	OD2	1	96.9
THR572	OG1	1	ASP570	O	1	87.5
SER967	OG	2	ASP570	O	1	97.9
THR572	N	3	ASP570	OD1	3	97.9
THR572	N	3	ASP570	OD2	3	89.6
LYS854	NZ	3	ASP570	OD2	2	94.8
GLN607	NE2	1	HIS681	O	1	99.0
ALA684	N	2	HIS681	O	2	87.5
SER673	OG	3	HIS681	ND1	3	90.6
THR676	OG1	3	HIS681	O	3	89.6
ASN717	ND2	3	ILE716	O	3	100.0
ARG1091	NH1	1	HIS1118	O	2	92.7
ARG1091	NH2	1	HIS1118	O	1	97.9
ARG1091	NH2	1	HIS1118	O	2	99.0
ARG1091	NH2	2	HIS1118	O	3	100.0
HIS1118	NE2	2	HIS1118	ND1	1	92.7
THR1116	OG1	3	HIS1118	N	3	91.7

Table S3. Occupancy (% residence time) of the main hydrogen bonds between the glycan linked to N165, N122 and N149 of monomer 3 and the amino acids of RBD of monomer 2 in up conformation in the S protein WT. For the glycan at N234 (monomer 3), the occupancy of the intra-monomer hydrogen bonds with the amino acids of the RBD (monomer 3) are reported.

Glycan	Residue	% residence time
N122	THR333	6.9
	ASN334	32.9
	LEU335	26.0
	CYS336	2.2
	PRO337	2.9
	GLU340	31.0
N149	GLU340	74.9
	PHE342	2.2
	ASN343	100.0
	ALA344	10.4
	THR345	72.0
	ARG346	14.5
	ARG509	3.0
N165	ARG357	34.96
	ASN394	15.3
	LYS462	17.2
	GLU465	12.15
	GLU516	81.8
	LEU517	7.7
	LEU518	2.4
	HIS519	15.9
N234	SER366*	2.9
	TYR369*	71.8
	ASN370*	40.1
	THR385*	71.5
	ASN388*	89.9
	ASP389*	100.0
	LYS528*	16.1

*Amino acids of RBD domain of monomer 3

Table S4. Occupancy (% residence time) of the hydrogen bonds between the glycan at N165 and N234 of the monomer 2 and between the glycans linked to N122 and N149 of the monomer 3 and the glycan at N331 and N343 of monomer 2 (up conformation).

Glycan pairs	% residence time	
	WT	UK
N165[a]-N234[a]	83 .4	97.0
N122[a]-N331[b]	94.5	87.0
N122[a]-N343[b]	50.4	96.9
N149[a]-N331[b]	1.3	0
N149[a]-N343[b]	32.1	97.3

[a] monomer 2

[b] monomer 3

Table S5. Occupancy (% residence time) of the hydrogen bonds between the glycan linked to N122, N149, N165 and N234 of the monomer 3 and the amino acids of RBD domains of monomer 2 (up conformation) and monomer 3.

**Glycan-RBD pairs**	**% residence time**	
	**WT**	**UK**
N122-RBD_2 ("up")	55.0	19.5
N149-RBD_2 ("up")	93.9	0.0
N165-RBD_2 ("up")	96.7	14.64
N165-RBD_3	79.2	10.2
N234-RBD_3	99.8	98.2

Table S6. Occupancy (% residence time) of the main hydrogen bonds between the glycan linked to N165 and N122 of monomer 3 and the amino acids of RBD of monomer 2 in up conformation in the S protein UK. For the glycan at N234 (monomer 3), the occupancy of the intra-monomer hydrogen bonds with the amino acids of the RBD (monomer 3) are reported.

**Glycan**	**Residue**	**% residence time**
N122	THR333	4.8
	ASN334	3.6
	LEU335	5.0
	GLU339	11.0
N165	ARG357	9.2
	ASN396	4.0
N234	ASP361*	68.7
	THR362*	6.3
	SER363*	9.2
	TYR366*	29.7
	THR382*	29.0
	ASN385*	19.4
	ASP386*	78.7
	LYS525*	12.9
	LYS526*	12.9

*Amino acids of RBD domain of monomer 3

Table S7. Site specific glycosylation in the model of SARS-CoV-2 S protein WT and UK mutant.

Residue	Monomer 1	Monomer 2 ("up")	Monomer 3
N61	M5	M5	M5
N74	A3	FA3G3S2	A2
N122	M5	FA2	M5
N149	FA2G2S1	FA3	FA2
N165	FA2G2S2	M5	FA2G2S1
N234	M8	M9	M9
N282	FA3	FA3G3S1	A2
N331	FA2	FA2	FA3G3S1
N343	FA2	FA1	FA2
N603	FA2	M5	M5
N616	A2	FA2	FA2
N657	M5	Hybrid G1	Hybrid G1
N709	M6	M5	M5
N717	Hybrid G1	M5	M6
N801	M6	M7	M5
N1074	FA2G2S1	M5	M5
N1098	FA2	A2	Hybrid G1S1
N1134	FA1	FA3	FA2
T323	O-glycan[a]	O-glycan[b]	O-glycan[c]
S325	O-glycan[a]		

[a] DNeu5Aca2-3DGalb1-3DGalNAca1-OH

[b] DNeu5Aca2-3DGalb1-3[DNeu5Aca2-6]DGalNAca1-OH

[c] DGalb1-3DGalNAca1-OH

References

1. Tagliamonte, M.S.; Abid, N.; Borocci, S.; Sangiovanni, E.; Ostrov, D.A.; Kosakovsky Pond, S.L.; Salemi, M.; Chillemi, G.; Mavian, C. Multiple recombination events and strong purifying selection at the origin of SARS-CoV-2 spike glycoprotein increased correlated dynamic movements. *Int. J. Mol. Sci.* **2021**, *22*, 1–16.
