# Supplementary figures and images for: Altered Local Interactions and Long-Range Communications in UK Variant (B.1.1.7) Spike Glycoprotein
Source: Int J Mol Sci. 2021 May 22;22(11):5464. doi: 10.3390/ijms22115464 (PMC8196891; doi:10.3390/ijms22115464)

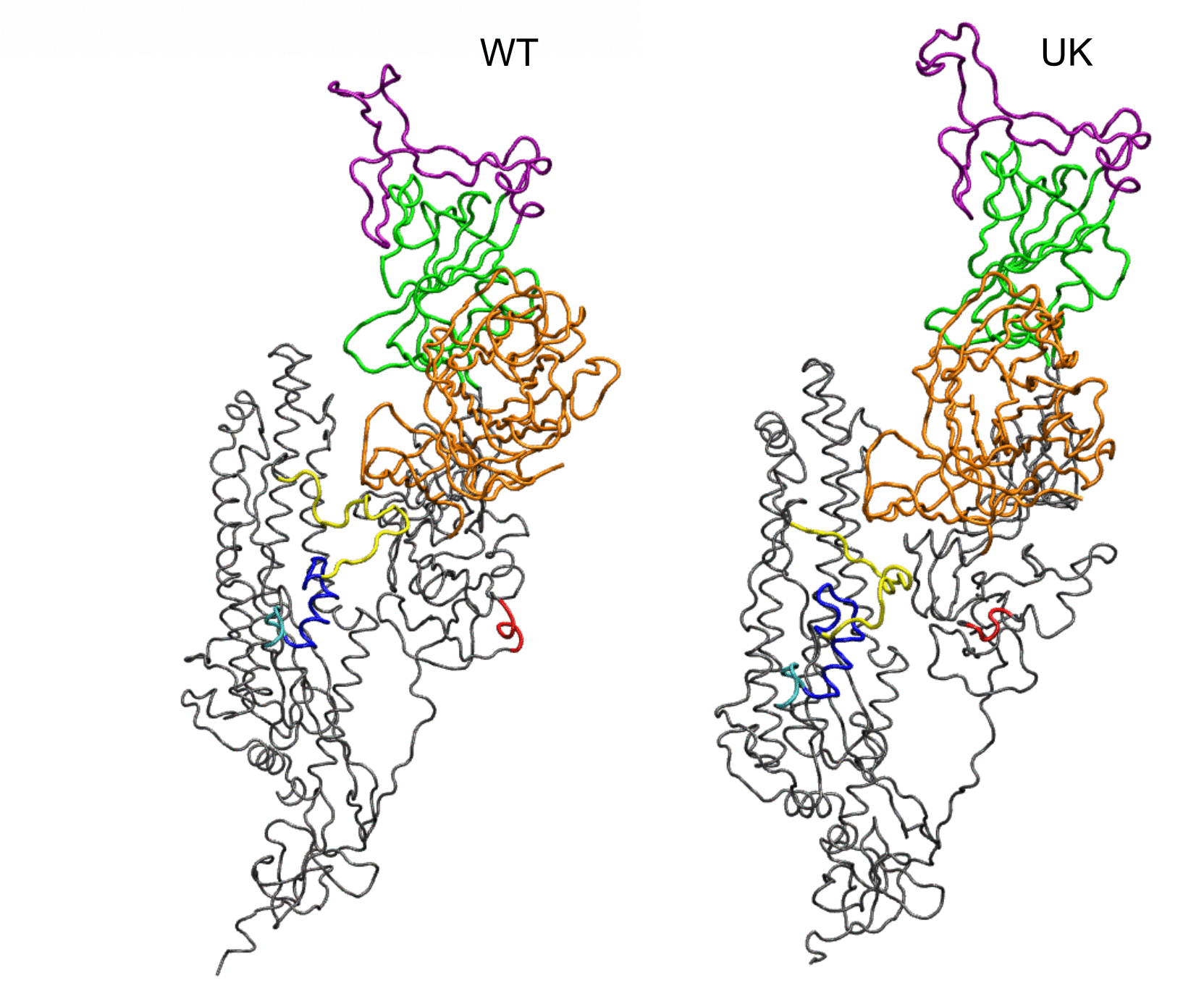

Supplement: Supplementary file 1 [file ijms-22-05464-s001.zip › Supplementary_Materials/movie_1.gif]

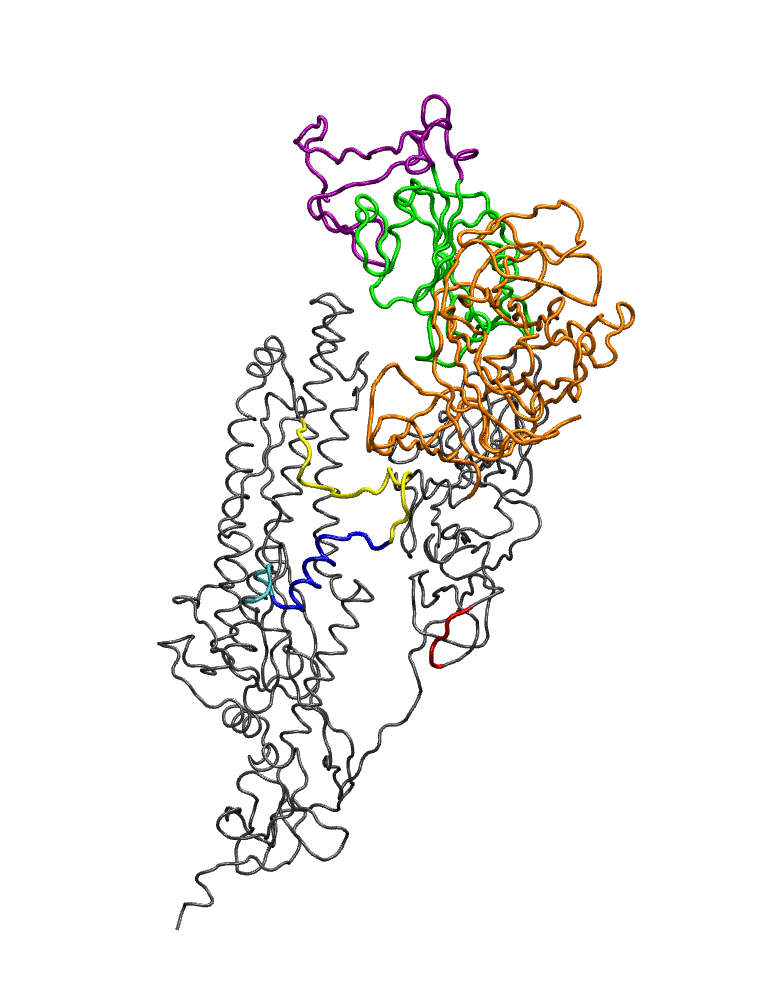

Supplement: Supplementary file 1 [file ijms-22-05464-s001.zip › Supplementary_Materials/movie_2.gif]

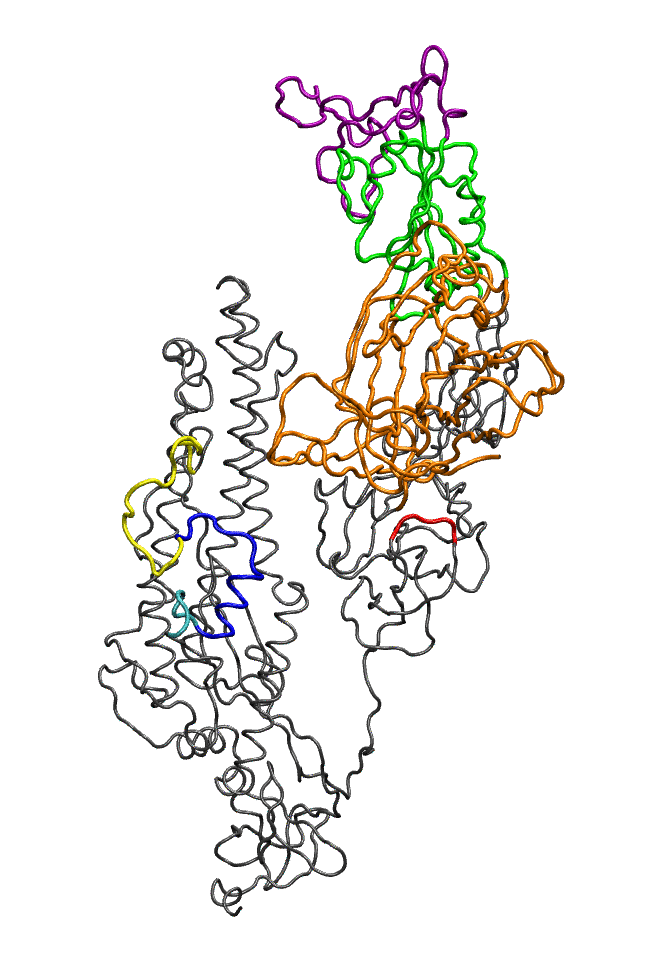

Supplement: Supplementary file 1 [file ijms-22-05464-s001.zip › Supplementary_Materials/movie_3.gif]
